# Deep Learning-Assisted Diagnostic System: Apices and Odontogenic Sinus Floor Level Analysis in Dental Panoramic Radiographs

**DOI:** 10.3390/bioengineering12020134

**Published:** 2025-01-30

**Authors:** Pei-Yi Wu, Yuan-Jin Lin, Yu-Jen Chang, Sung-Tsun Wei, Chiung-An Chen, Kuo-Chen Li, Wei-Chen Tu, Patricia Angela R. Abu

**Affiliations:** 1 Department of Periodontics, Division of Dentistry, Taoyuan Chang Gung Memorial Hospital, Taoyuan City 333034, Taiwan; q384@cgmh.org.tw; 2 Department of Program on Semiconductor Manufacturing Technology, Academy of Innovative Semiconductor and Sustainable Manufacturing, National Cheng Kung University, Tainan City 701401, Taiwan; m28121562@gs.ncku.edu.tw; 3 Department of Electronic Engineering, Chung Yuan Christian University, Taoyuan City 32023, Taiwan; s11126110@cycu.edu.tw (Y.-J.C.); s11126121@cycu.edu.tw (S.-T.W.); 4 Department of Electrical Engineering, Ming Chi University of Technology, New Taipei City 243303, Taiwan; 5 Department of Information Management, Chung Yuan Christian University, Taoyuan City 32023, Taiwan; 6 Department of Electrical Engineering, National Cheng Kung University, Tainan City 701401, Taiwan; wctu@gs.ncku.edu.tw; 7 Ateneo Laboratory for Intelligent Visual Environments, Department of Information Systems and Computer Science, Ateneo de Manila University, Quezon City 1108, Philippines; pabu@ateneo.edu

**Keywords:** image enhancement, intelligent healthcare, medical image processing, object detection, odontogenic sinusitis, YOLOv8n-cls, YOLO 11n

## Abstract

Odontogenic sinusitis is a type of sinusitis caused by apical lesions of teeth near the maxillary sinus floor. Its clinical symptoms are highly like other types of sinusitis, often leading to misdiagnosis as general sinusitis by dentists in the early stages. This misdiagnosis delays treatment and may be accompanied by toothache. Therefore, using artificial intelligence to assist dentists in accurately diagnosing odontogenic sinusitis is crucial. This study introduces an innovative odontogenic sinusitis image processing technique, which is fused with common contrast limited adaptive histogram equalization, Min-Max normalization, and the RGB mapping method. Moreover, this study combined various deep learning models to enhance diagnostic accuracy. The YOLO 11n model was used to detect odontogenic sinusitis single tooth position in dental panoramic radiographs and achieved an accuracy of 98.2%. The YOLOv8n-cls model diagnosed odontogenic sinusitis with a final classification accuracy of 96.1%, achieving a 16.9% improvement over non-enhanced methods and outperforming recent studies by at least 4%. Additionally, in clinical applications, the classification accuracy for non-odontogenic sinusitis was 95.8%, while for odontogenic sinusitis it was 97.6%. The detection method developed in this study effectively reduces the radiation dose patients receive during CT imaging and serves as an auxiliary system, providing dentists with reliable support for the precise diagnosis of odontogenic sinusitis.

## 1. Introduction

In recent years, the rapid development of artificial intelligence (AI) has brought significant advancements to various technological fields, and the medical domain is no exception. Particularly in integrating medical imaging and deep learning, AI has demonstrated its advantages in efficient learning, driving innovations in intelligent healthcare systems that surpass traditional methods [1,2,3,4]. These research findings underscore AI’s extensive applications and remarkable effectiveness in the medical field, providing a solid foundation for the continued development of intelligent healthcare. Moreover, in the field of AI-assisted dentistry, AI has shown significant potential in improving diagnostic efficiency and accuracy. Studies have demonstrated using CNN models to tackle various dental challenges, such as detecting retained roots, endodontic-treated teeth, implants, impacted third molars, apical lesions, caries, and restorations [5,6,7,8]. For instance, CNN-based models like ResNet, YOLOv3, AlexNet, and GoogleNet have achieved high accuracy levels, often exceeding 90%. This integration of AI into dental medicine enhances diagnostic precision and streamlines clinical workflows, addressing the growing demand for efficient and reliable dental care solutions. However, research specifically focusing on the application of AI to odontogenic sinusitis-related features remains scarce. This highlights a critical gap in leveraging AI to assist in diagnosing and managing this condition, which is often associated with complex dental and sinus interactions. Bridging this gap could significantly improve early detection and treatment planning for odontogenic sinusitis, benefiting both patients and practitioners.

Odontogenic sinusitis is a multifactorial disease that has garnered significant attention [9], with etiologies including dental surgeries, foreign bodies, and odontogenic infections [10]. Studies have shown a positive correlation between the increase in dental surgeries and the incidence of odontogenic sinusitis [11]. This condition predominantly affects individuals aged 40 to 60, with a slightly higher prevalence in females than males. In odontogenic infections, the infection often originates from diseased teeth, such as pulpitis or periodontitis. Bacteria spread through the dental pulp or periodontal tissues, extending along the tooth roots into the sinus cavity, causing sinus inflammation and infection. When the root apices are in close proximity to the sinus floor, periodontal disease, dental caries, or dental treatments such as extractions and implant procedures can further increase the risk of sinus infection. Improper postoperative wound care following tooth extraction may also lead to sinus membrane perforation, which occurs when the thin maxillary sinus mucosa or Schneiderian membrane is damaged, allowing direct communication between the oral cavity and the sinus. This condition predisposes patients to secondary infections and can result in oroantral communication, where air or fluids pass between the oral cavity and the sinus, leading to prolonged healing or surgical complications.

The early symptoms of odontogenic sinusitis closely resemble those of general sinusitis, including nasal congestion, rhinorrhea, and foul odor [12]. Due to these nonspecific symptoms, distinguishing odontogenic sinusitis from other types of sinusitis can be challenging. Diagnosis often requires collaboration between otolaryngologists and dental professionals [13], further complicating early detection. Moreover, only about one-third of patients experience dental pain, making it even more challenging to identify the condition promptly [14]. Recognizing the anatomical relationship between the apical part of the teeth and the sinus floor is crucial in diagnosing odontogenic sinusitis, as this knowledge enables more precise differentiation from other forms of sinusitis and facilitates timely intervention. Research indicates that approximately 70.4% of patients resolve their symptoms within six months, while only 11.1% experience symptoms for over a year [12]. However, if left untreated, odontogenic sinusitis can spread to other areas, posing more significant risks to the patient’s health. A key characteristic of this disease is the proximity of the affected tooth roots to the maxillary sinus floor. When diagnosing sinusitis, it is essential to consider the presence of diseased teeth with roots near the sinus floor, as this can lead to a quicker and more accurate diagnosis of odontogenic sinusitis. The clinical challenge in diagnosing odontogenic sinusitis lies in the fact that patients often focus on nasal symptoms, which can lead to misdiagnosis, especially in medically underdeveloped areas, resulting in delayed treatment.

In the current literature, most studies use computed tomography (CT) to detect odontogenic sinusitis [15]. However, traditional CT scans typically involve higher radiation doses. Nonetheless, modern dental CT equipment, such as cone-beam CT (CBCT), has significantly reduced radiation exposure and has become a routine tool in dental imaging, especially for handling cases related to odontogenic sinusitis [16]. On the other hand, dental panoramic radiographs (DPR) offer an effective alternative. They can extract diagnostic information that would typically require CT, thereby avoiding the need for CT scans, reducing radiation exposure, and retaining sufficient diagnostic information. Rather than solely diagnosing odontogenic sinusitis using DPR, this study focuses on utilizing DPR to preliminarily assess whether the apical part of the teeth is close to the sinus floor. This early evaluation helps prevent potential odontogenic sinusitis or sinus membrane perforation during dental treatments. Furthermore, it allows for appropriate preventive measures, such as applying collagen after tooth extraction, to minimize the risk of sinus membrane perforation. To address challenges related to radiation exposure and diagnostic costs, this study employs image enhancement and deep learning techniques to process odontogenic sinusitis-related features from DPR. These approaches aim to enhance diagnostic performance, minimize radiation risks for patients, and assist dentists in preliminary diagnosis and treatment [17].

This study aims to develop an automated diagnostic system using image processing and deep learning techniques. The system is designed to determine whether dental root apices are close to the sinus floor. Additionally, it highlights sinus locations through image enhancement. This technology instantly identifies the proximity between tooth roots and the sinus floor when capturing a DPR image. It alerts patients to potential risks and facilitates case information sharing with otolaryngologists, providing additional reference data for clinical diagnosis. Moreover, the technology is applicable in resource-limited areas, aiding in screening high-risk populations and enhancing early diagnostic capabilities. In recent years, research combining artificial intelligence and medical imaging for automated diagnosis has gained significant attention [18]. Many studies have utilized image segmentation techniques on DPR images to enhance lesion recognition accuracy and optimize diagnostic results. To further strengthen model performance, this study applied a series of image preprocessing techniques to single-tooth images, including Gaussian blur [19], grayscale conversion [20], Contrast Limited Adaptive Histogram Equalization (CLAHE) [21], min–max normalization, and RGB mapping [22]. By advancing AI applications in medical imaging, this study seeks to address the limitations of current diagnostic methods in resource-constrained environments.

## 2. Materials and Methods

**Figure 1:**
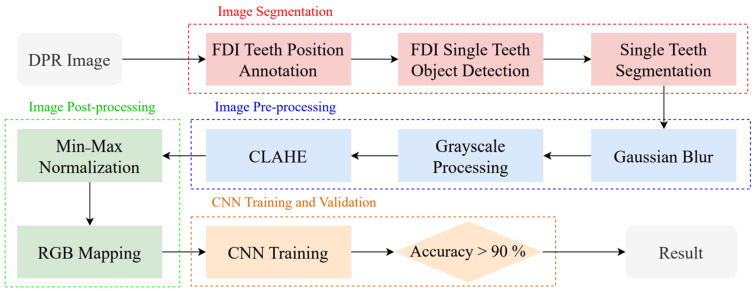
Assisted evaluation of odontogenic sinusitis research flowchart.

To enable lesion identification, this study adopted the workflow illustrated in Figure 1. First, DPR images were used as input and processed through a two-stage image segmentation technique to generate a dataset for training single-tooth detection. Subsequently, various image processing techniques were applied, and five different classification models were used for cross-comparison. Finally, the results were evaluated for the diagnosis of odontogenic sinusitis.

### 2.1. Single Tooth Object Detection

**Figure 2:**
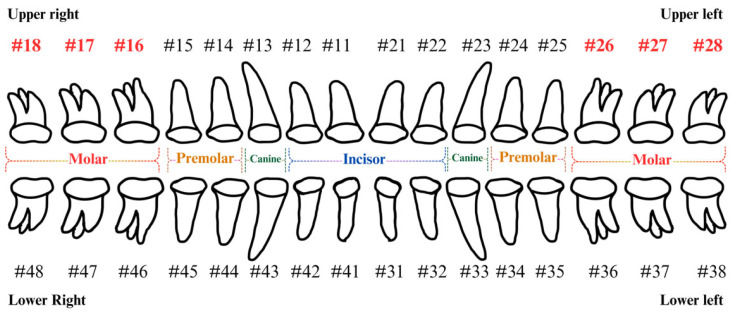
Fédération Dentaire Internationale tooth position representation.

In this study, the maxillary region of each DPR image was annotated at the far-left and far-right sides, with the marked positions defined as the Region of Interest (ROI) for sinus invasion lesions, referred to as ROI-I. This region includes teeth #16, #17, #18, #26, #27, and #28, based on the Fédération Dentaire Internationale (FDI) numbering system [23], as shown in Figure 2. Single-tooth regions were further segmented from ROI-I to train the final classification model, defining them as ROI-II. This step aims to generate the dataset required for model training, enabling the model to be trained in more refined regions to enhance classification performance and accuracy.

#### 2.1.1. ROI-I and ROI-II Annotation

**Figure 3:**
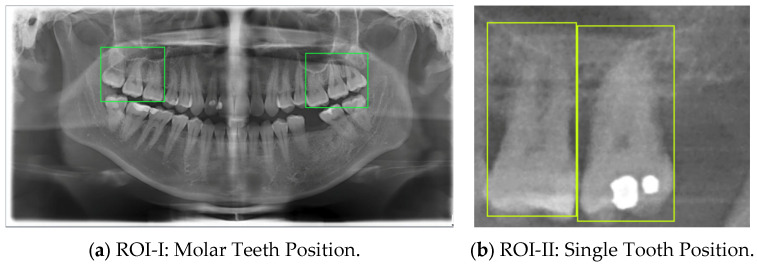
DPR image annotation.

Since the lesion areas near the sinus floor are located at the posterior left and right maxillary regions, it is essential to segment the Region of Interest (ROI) from DPR images before conducting lesion analysis. First, ROI-I includes teeth #16, #17, #18, #26, #27, and #28 based on the Fédération Dentaire Internationale (FDI) numbering system. This region is selected using quadrilateral bounding boxes, as illustrated in Figure 3a. This process ensures that ROI-I effectively encompasses potential sinus invasion lesion sites while excluding irrelevant areas, thereby improving the accuracy of subsequent model segmentation. After obtaining ROI-I, individual tooth regions (ROI-II) are further segmented. The precise quadrilateral tool is used for marking, ensuring the inclusion of the sinus line and the single tooth, as shown in Figure 3b. This method accurately captures the alignment and position of lesions, providing high-quality input data for CNN model segmentation and analysis. This two-stage segmentation approach significantly improves the accuracy of detecting whether tooth roots are close to the sinus floor in cases of odontogenic sinusitis, offering more reliable technical support for lesion analysis and assisted diagnosis.

#### 2.1.2. Object Detection Model

**Figure 4:**
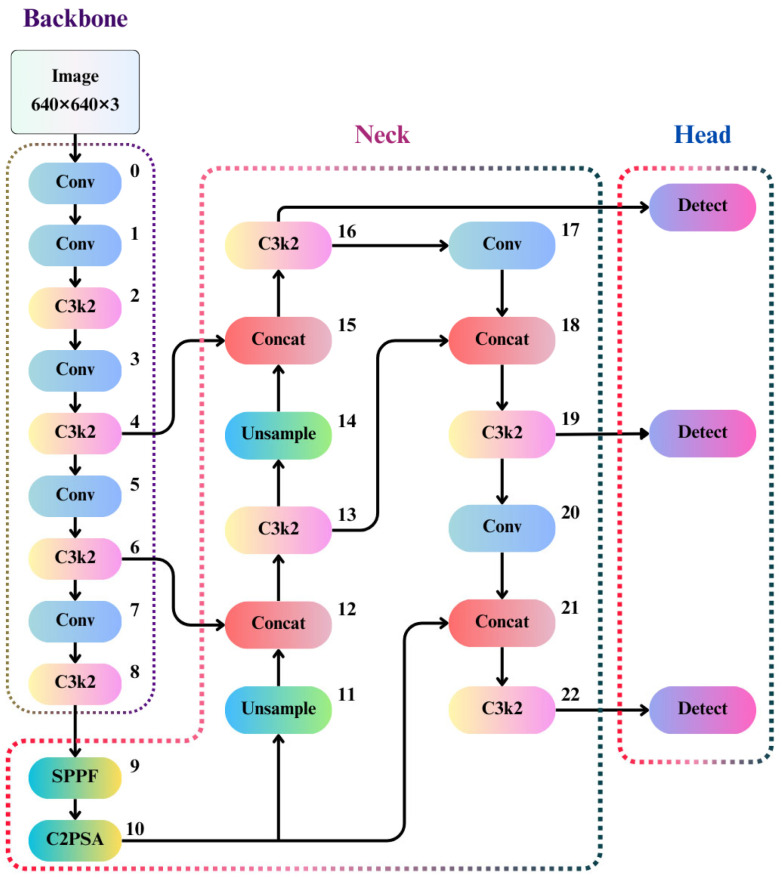
YOLO11n block diagram.

This study conducted image detection and analysis for the segmented regions ROI-I and ROI-II, processing them for different targets. ROI-I was used to detect potential lesion areas in DPR images, while ROI-II focused on precisely detecting individual teeth and surrounding details. A CNN model was chosen as the foundational framework for initial training. To ensure optimal recognition performance in the segmented regions, various YOLO-based object detection architectures, including YOLOv8n, YOLOv9n, YOLOv10n, and YOLO11n, were comprehensively compared. Each model has unique features, and YOLO11n was selected as the primary framework for this study, and the block diagram and architecture are shown in Figure 4 and Table 1. YOLO11n integrates advanced technologies, including an upgraded feature extraction module, an improved multi-scale detection mechanism, and an optimized lightweight architecture, achieving a remarkable balance between speed and accuracy. Compared to YOLOv8n, YOLO11n reduces the number of parameters by 22% and is 2% faster in inference speed than YOLOv10n. Moreover, YOLO11n is optimized explicitly for Oriented Object Detection, enhancing the model’s ability to identify rotated or non-axis-aligned objects. In the YOLOv8n architecture, the C2f module optimizes the training process by introducing residual connections. YOLO11n replaces the C2f module with the C3k2 module, which uses two smaller convolutional layers combined with multi-path feature fusion techniques. This replacement accelerates data processing, reduces computational load, and improves feature extraction efficiency. Independent image datasets were prepared for image detection in ROI-I and ROI-II, with their distribution detailed in Table 2. ROI-I consisted of 163 panoramic PA images, while ROI-II was derived from ROI-I, comprising 129 images. These datasets were divided into training, validation, and test sets in a 7:2:1 ratio to ensure the reliability and stability of model training and evaluation.

### 2.2. Single Tooth Image Preprocessing

In the early stages of model training, image preprocessing plays a crucial role in enhancing image features, directly affecting the stability and final performance of the model. This study adopted three preprocessing methods, Gaussian blur, grayscale processing, and CLAHE, to improve overall image quality and optimize feature extraction. Gaussian blur effectively reduces high-frequency noise in images by smoothing out texture details, thereby highlighting the overall contours of the target region. CLAHE enhances local contrast and addresses uneven brightness distribution in images, ensuring that detailed features in DPR images are visible under varying brightness conditions. The combination of these preprocessing methods not only provides the model with a stable and high-quality input dataset, but also improves model accuracy, laying a solid foundation for subsequent training and evaluation.

#### 2.2.1. Gaussian Blur

Tooth images often contain significant noise, which can reduce the effectiveness of deep learning training. To address this issue, this study applies Gaussian blur to grayscale images for noise reduction, thereby smoothing the image by reducing details. Each pixel is calculated as a weighted average of its surrounding pixels, achieving overall image smoothing, and the formula is shown in (1):

(1) $$G(x,y)=\frac{1}{2\pi \sigma^{2}}e^{-\frac{x^{2}+y^{2}}{2\sigma^{2}}}$$

**Figure 5:**
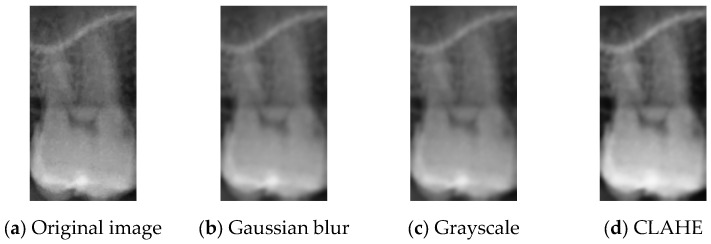
Single tooth image preprocessing.

The degree of blurring is controlled by adjusting the standard deviation σ. A larger σ value results in a more pronounced blurring effect. The symbols x and y represent the horizontal and vertical distances from the center of the kernel to the pixel being processed, respectively. The before and after Gaussian Blur results are shown in Figure 5a,b.

#### 2.2.2. Grayscale Processing

This study employed the weighted averaging method to transform the red, green, and blue (R, G, B) channel values of color images into a single grayscale value, as the formula in (2) describes. The weights in this formula are derived from the equation specified by the National Television System Committee (NTSC) for wide-band luminance signals, as referenced in [24].

(2) Gray=0.299R+0.587G+0.114B

The primary purpose of grayscale processing is to simplify the data structure of images by removing color information and compressing three-channel data into a single channel. This significantly reduces computational complexity and resource consumption in subsequent processing. This method preserves the image’s main structural features and brightness information, as illustrated in Figure 5c. Grayscale processing enhances the contrast of lesion areas in dental medical imaging, making critical structures more prominent and further improving the model’s recognition capabilities. Compared to the simple averaging method, the weighted averaging method used in this study more accurately restores the brightness characteristics of the image, thereby improving the precision of image processing.

#### 2.2.3. Contrast Limited Adaptive Histogram Equalization Process

This study also applied Contrast Limited Adaptive Histogram Equalization (CLAHE), with the processing results shown in Figure 5d. CLAHE performs histogram equalization within local regions while limiting contrast enhancement by clipping the histogram. It prevents the over-enhancement issues often associated with traditional adaptive histogram equalization. Before calculating the cumulative distribution function (CDF), the histogram is clipped to ensure that pixel values exceeding the predefined threshold do not affect contrast adjustment. This clipping effectively suppresses noise in the image, preventing it from being excessively amplified during the equalization process while preserving its overall structure and detailed features. In dental medical imaging and other low-contrast imaging applications, CLAHE significantly improves the visibility of details. Due to its localized processing characteristics, this technique enhances the contrast in critical regions while avoiding over-enhancement of the entire image. It provides higher-quality data for image analysis, contributing to improved diagnostic accuracy of the model.

### 2.3. Single Tooth Image Postprocessing

Image post-processing is a critical step in sinus floor lesion identification. This study found that in ROI-II, the min–max normalization result of grayscale areas is essential for diagnosing lesions. Therefore, the pixel values of ROI-II were first analyzed to calculate min–max normalization, capturing the distribution characteristics of grayscale levels. Subsequently, using RGB mapping techniques, the grayscale X-ray images were converted into colored images, with different grayscale regions colorized to highlight the relative relationship between the sinus and teeth.

#### 2.3.1. Min–Max Normalization

**Figure 6:**
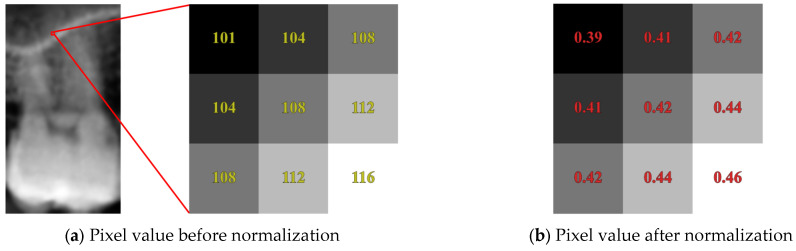
Min–max normalization analysis of single tooth images.

The original single teeth image contains pixel values ranging from 0 to 255, as shown in Figure 6a. During deep learning training, the wide range of pixel values may lead to instability in the training process and even cause gradient explosion. To mitigate these issues, this study employs the Min-Max normalization method to scale each feature linearly to the range of [0,1], as described by Equation (3), where p represents the pixel value of the image. Normalized data not only prevent certain pixel values from disproportionately influencing the model but also effectively reduce the magnitude differences in the training data, thereby improving training efficiency. The pixel values after normalization are illustrated in Figure 6b.

(3) $$p’=\frac{p-min(p)}{\max\left(p\right)-min(p)}$$

#### 2.3.2. RGB Mapping

**Figure 7:**
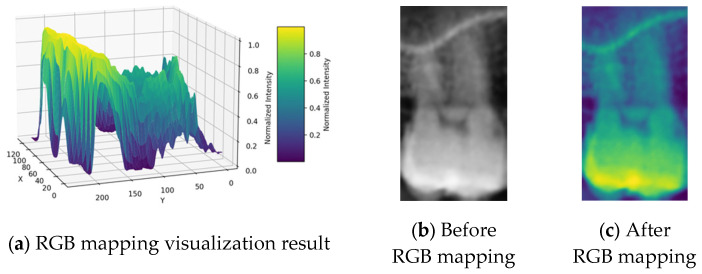
RGB mapping processing.

RGB mapping is a visualization technique that assigns colors to intensity values in 2D images, thereby highlighting regions of interest. In this study, after normalizing the data, the RGB mapping technique was applied to map the values onto the *z*-axis dimension, with the visualization results shown in Figure 7a. This approach effectively enhanced the contrast between the dental apices and the sinus, with the results before and after processing presented in Figure 7b,c. This process provided the model with more distinguishable image inputs, thereby improving its ability to identify pathological features. Furthermore, this study utilized RGB mapping to represent continuous color transitions for data features while ensuring clear differentiation between distinct color regions, further enhancing the visualization of image characteristics. The normalized value v was converted into the RGB color space using Equation (4), where R, G, and B represent the mapping functions for the red, green, and blue channels. The R, G, and B functions were calculated based on the value v using Formulas (5)–(7).

(4) $$Color\left(v\right)=(R\left(v\right),G\left(v\right),B\left(v\right))$$

(5) $$R\left(v\right)=Int(71-34\cdot v+541\cdot v^{2}-3684\cdot v^{3}+6300\cdot v^{4}-2943\cdot v^{5})$$

(6) $$G\left(v\right)=Int(408-479\cdot v+677\cdot v^{3}-432\cdot v^{4}+44\cdot v^{5})$$

(7) $$B\left(v\right)=Int(77+652\cdot v-2998\cdot v^{2}+7226\cdot v^{3}-8373\cdot v^{4}+3441\cdot v^{5})$$

These formulas transformed v into RGB color space, where the operation Int was applied to convert formula outputs into integers. The R, G, and B functions primarily represent the data with continuous color transitions ranging from blue to green to yellow. This transition effectively visualizes variations, making it easier to distinguish subtle differences.

### 2.4. Disease Classification Model and Classification Dataset

Convolutional Neural Networks (CNN) have demonstrated outstanding performance in the field of image classification. Their core architecture consists of convolution layers, pooling layers, and fully connected layers. By leveraging a multi-layer structure, CNNs can effectively capture key features in images and are widely applied in fields such as audio and image classification. With their robust feature extraction capabilities, CNNs have become a mainstream method for training image-based models.

#### 2.4.1. Odontogenic Sinusitis Classification Model

In this study’s automated root invasion sinus recognition system, this study focused on CNN models and Transformer-based architectures, both of which have shown exceptional performance in image classification tasks. CNNs have long been used in medical imaging analysis for their superior ability to extract local features. Their core mechanism involves convolutional operations to capture fine-grained image features while significantly reducing computational costs and model complexity through parameter sharing.

This study compared five classification models: ResNet18 [25], ConvNeXtV2 [26], YOLOv8n-cls [27], YOLO11n-cls [28], and Swin-Transformer [29]. The characteristics and advantages of each model are outlined below. Moreover, YOLOv8n-cls and ResNet-18 are more commonly used in clinical practice, and the combined performance of these two models is significantly better than other models. Therefore, this study focuses on the application of these two models in symptom analysis.

1. ResNet18

**Figure 8:**
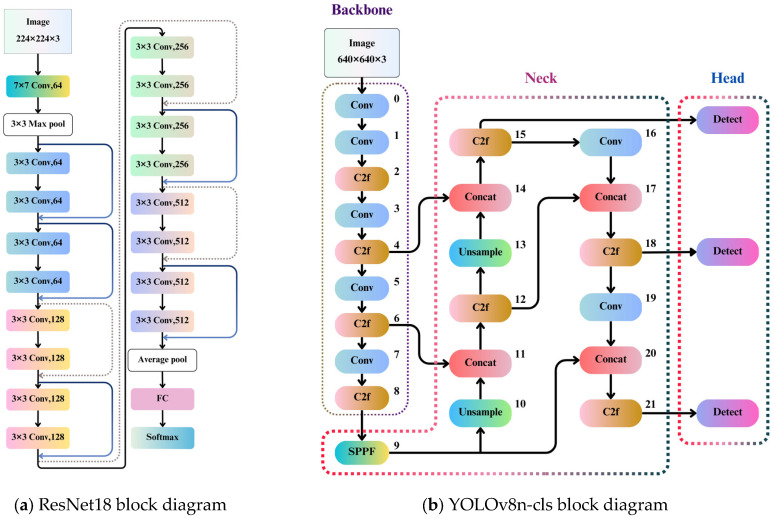
Fine-tuning classification model used in this study.

ResNet18 introduced residual connections to address the vanishing gradient problem in deep networks. It has demonstrated stable performance across various classification tasks; the block diagram and architecture are shown in Figure 8a and Table 3.

2. ConvNeXtV2

ConvNeXtV2 represents a modernized convolutional neural network (ConvNet) architecture, combining the strengths of self-supervised learning and ConvNet design. This model incorporates the Fully Convolutional Masked Autoencoder (FCMAE) framework and Global Response Normalization (GRN) techniques to enhance performance in tasks such as image classification, object detection, and semantic segmentation.

3. YOLOv8n-cls and YOLO11n-cls

The YOLO series is renowned for object detection; its lightweight design and optimized classification head also enable strong performance in classification tasks. YOLO11n-cls further introduces an improved multi-scale feature detection mechanism and a lightweight architecture, achieving a notable balance between speed and accuracy; the YOLOv8n-cls block diagram and architecture are shown in Figure 8b and Table 4.

4. Swin-Transformer

The Swin-Transformer is a representative Transformer-based model, which uses a hierarchical structure and shifted window attention mechanism to reduce computational complexity effectively while capturing multi-scale features. This makes it particularly suitable for analyzing high-resolution medical images.

This study utilized the NVIDIA A100 40G GPU for high-performance acceleration to ensure the efficiency and accuracy of model training and validation. The A100 offers powerful computational capabilities and large memory capacity, enabling support for large-scale datasets and deploying complex models. The hardware and software platform configurations used in this study are detailed in Table 5.

#### 2.4.2. Classification Model Training and Validation and Dataset

Data processing and allocation are critical steps in the training and evaluation of machine learning models. This study adopted the following two strategies for the single-tooth image dataset. First, the original dataset was divided into two parts, as shown in Table 6, with 90% used for model training and 10% for validation. This allocation ensures the independence of the training process while providing sufficient validation data to evaluate model performance. However, due to the limited size of the original dataset, the model’s generalization capability may need to be revised. Data augmentation techniques were applied to address the issue of insufficient data. Specific methods included random rotation of the original images (within a range of ±5 degrees), adding random noise, and adjusting brightness. These techniques expanded the dataset to approximately three times its original size, significantly enhancing the model’s generalization capability. The distribution of the augmented dataset is shown in Table 7.

## 3. Results

This section is divided into two subsections, beginning with the detection results of single-tooth regions in DPR images, followed by an analysis of lesion classification models and their comparative evaluation across various metrics. This study used five metrics to evaluate the results, as shown in (8)–(12), and selected for analysis to comprehensively assess model performance: accuracy, precision, recall, F1 Score, and mAP50. These metrics were used to quantify the models’ classification capabilities and detection efficiency. Each formula is explained below:

1. Accuracy: Measures the proportion of correct predictions among all samples.
2. Recall: Assesses the model’s ability to identify all actual positives.
3. Precision: Indicates the proportion of true positives among predicted positives.
4. F1-Score: Balances Precision and Recall, suitable for imbalanced datasets.
5. mAP: Averages the precision across all classes, commonly used in detection tasks.

(8) $$Accuracy=\frac{T_{P}+T_{N}}{T_{P}+T_{N}+F_{P}+F_{N}}$$

(9) $$Recall=\frac{T_{P}}{T_{P}+F_{N}}$$

(10) $$Precision=\frac{T_{P}}{T_{P}+F_{P}}$$

(11) $$F1\;\mathrm{Score}=2\times \frac{Precision\times Recall}{Precision+Recall}$$

(12) $$mAP=\frac{1}{n}\sum_{i=1}^{n}AP_{i}$$

In the context of evaluation metrics, these notations are fundamental components used to assess model performance. True Positive (T_P_) represents instances correctly identified as belonging to the positive class, while True Negative (T_N_) indicates instances correctly classified as belonging to the negative class. False Positive (F_N_) refers to cases incorrectly predicted as positive when they belong to the negative class. False Negative (F_N_) denotes instances wrongly classified as unfavorable when they genuinely belong to the positive class. In calculating mean Average Precision (mAP), n represents the total number of classes or queries being evaluated, and AP_i_ specifically denotes the Average Precision calculated for the i class or query in the evaluation set.

### 3.1. DPR Image Object Detection Result

Table 8 demonstrates a comparison of different YOLO model performances. First, YOLO11n has outstanding performance across all metrics in the ROI-I phase, achieving an accuracy of 90.0%. This represents a significant improvement compared to YOLOv8n (70.6%) and YOLOv10n (80.0%) while being slightly higher than YOLOv9n (89.5%). Its precision reached 94.4%, the highest among all models, indicating that YOLO11n effectively minimizes false positive results. The recall was also 94.4%, matching YOLOv9n’s performance. In molar teeth position detection, the image shows two highlighted regions with prediction accuracies of 92% and 91%, indicating consistent performance in molar teeth detection. In the ROI-II phase, YOLO11n achieved the best performance, with an accuracy of 93.2%, precision of 94.8%, and a remarkably high recall of 98.2%. For the single tooth position detection, the highlighted regions show prediction accuracies of 89% and 99%, demonstrating the model’s capability to precisely identify individual teeth. These metrics indicate that YOLO11n excels in classifying target regions and detecting all target instances. Additionally, its F1 score reached 98.2%, demonstrating exceptional capability in balancing precision and recall, further solidifying its advantage in detection and classification tasks.

**Figure 9:**
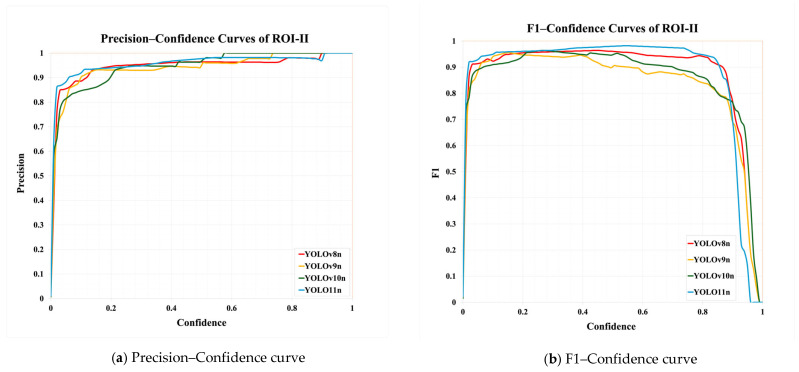
ROI-II detection using YOLO models.

In Figure 9a, the precision–confidence curve shows that YOLO11n outperforms other models in most confidence intervals, particularly in high-confidence regions where its curve displays a stable and precise trend. This reflects the model’s reliability in high-confidence intervals. Additionally, in Figure 9b, the F1-confidence curve illustrates that YOLO11n maintained a high F1 score across different confidence values, primarily excelling in the mid-to-high confidence range. This indicates that YOLO11n has significant advantages in balancing precision and recall, enabling more accurate identification of target regions while enhancing the overall reliability of detection.

### 3.2. Single Tooth Classification Model

This study compared five classification models: ResNet18, ConvNeXtV2, Swin-Transformer, YOLOv8n-cls, and YOLO11n-cls. During the evaluation of each model’s performance in the training and validation phases, particular attention was paid to the trend of the loss function curves to explore the models’ applicability and learning efficiency. The loss function, which measures the difference between the model’s predictions and the proper labels, is a core indicator. Its behavior during training and validation directly reflects the model’s learning effectiveness and stability. In the training phase, changes in the loss curve provide insights into the model’s convergence speed and stability on the dataset. Meanwhile, the validation phase loss reflects the model’s generalization performance on unseen data. If the loss curve stabilizes too early or if there is a significant deviation between the training and validation curves, it may indicate overfitting or underfitting issues. The loss function curves for YOLOv8n-cls and ResNet18 during both the training and validation phases are shown in this study. For classification tasks, the study employs the binary cross-entropy loss function, which is defined by Equation (13). This function calculates the difference between the predicted probability, $p\left(x_{i}\right)$, that an instance $x_{i}$ belongs to the default class, and the true binary label $y_{i}$, which can be either 0 or 1. This loss function computes the logarithmic error between the predicted probability and the true label for each instance. When the predicted probability $p\left(x_{i}\right)$ is close to the true label $y_{i}$, the loss is small, indicating a correct prediction.

(13) $$L=-(y_{i}\log\left(p\left(x_{i}\right)\right)+(1-y_{i})\log\left(1-p\left(x_{i}\right)\right))$$

**Figure 10:**
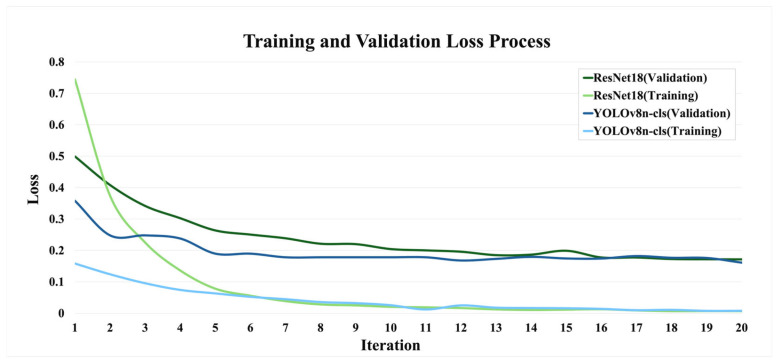
Loss function diagram during the training and verification process of YOLOv8n-cls and ResNet18.

From Figure 10, it is evident that YOLOv8n-cls demonstrates faster convergence in the training phase compared to ResNet18. The training loss for YOLOv8n-cls decreases more rapidly and stabilizes at a lower value, indicating higher learning efficiency and potentially better optimization during training. Additionally, the final validation loss for YOLOv8n-cls is lower than that of ResNet18, reinforcing its superior performance in handling unseen data. Overall, YOLOv8n-cls demonstrates better convergence speed, learning stability, and generalization compared to ResNet18 in this analysis.

Table 9 presents the impact of the image processing techniques developed in this study on model accuracy, including image preprocessing techniques (Gaussian Blur + Grayscale Processing + CLAHE) and postprocessing techniques (Min–Max normalization + RGB Mapping). The original dataset’s classification accuracies of ResNet18 and YOLO11n-cls were 85.7% and 90.9%, respectively. After applying preprocessing, the performance of nearly all models improved significantly, with the classification accuracy of Swin-Transformer increasing to 96.1%, indicating its higher sensitivity to images optimized for brightness distribution. The model performance was further enhanced when combining preprocessing and postprocessing techniques. YOLOv8n-cls achieved a classification accuracy of 96.1%, surpassing YOLO11n-cls and demonstrating the effectiveness of this combined processing approach in enhancing classification capabilities. Furthermore, after integrating all processing techniques, YOLOv8n-cls ultimately achieved a classification accuracy of 96.1% and a recall rate of 97.1%, representing a 16.9% improvement in accuracy compared to the original dataset.

**Figure 11:**
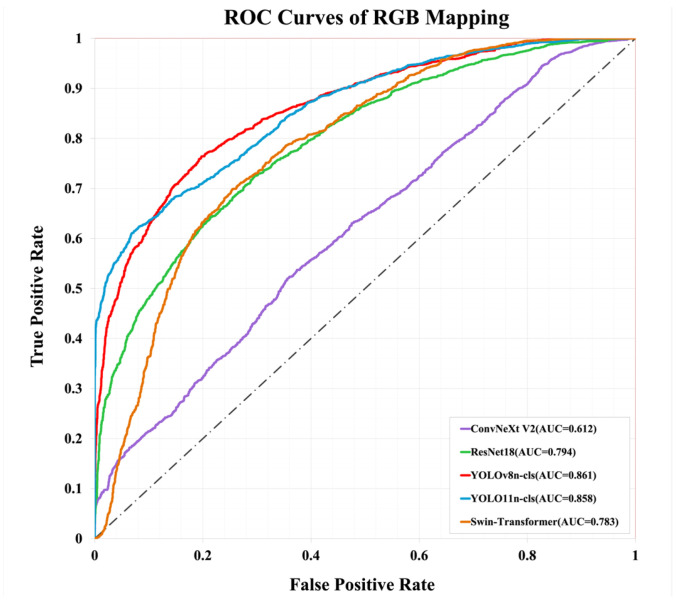
Comparison of the ROC curves of each model under RGB Mapping.

Additionally, this study conducted a detailed evaluation of the YOLOv8n-cls model’s performance in image classification tasks. From the ROC curve in Figure 11, it can be observed that YOLOv8n-cls achieved an AUC value of 0.861, significantly higher than ResNet18 (AUC = 0.794) and Swin-Transformer (AUC = 0.783). This highlights the outstanding classification accuracy of YOLOv8n-cls and its ability to maintain a good balance between the True Positive Rate and False Positive Rate.

From the confusion matrix data in Table 10, it is evident that the YOLOv8n-cls exhibited a high recognition capability and stability level when distinguishing whether a tooth root apex was near the sinus floor. For the category where the tooth root apex was not near the sinus floor, the model successfully identified 41 samples, with only one misclassification. In the category where the tooth root apex was near the sinus floor, the model correctly identified 33 samples, with just two misclassifications. These results demonstrate that the model achieves exceptionally high accuracy across different conditions.

Table 11 provides clinical assessment results. For cases without sinus invasion, YOLOv8n-cls achieved an average precision of 97.6%, outperforming ResNet18, which achieved 91.7%. Similarly, for cases involving sinus invasion, YOLOv8n-cls achieved a higher average precision of 95.8% compared to 90.5% for ResNet18. This indicates that YOLOv8n-cls possesses superior recognition capabilities for handling more complex pathological features. 

## 4. Discussion

This study proposed a deep learning-based solution for the automated diagnosis of whether tooth root apices are near the sinus floor, successfully integrating image processing techniques with CNN to identify such lesions in DPR images. The results demonstrated that through two-stage ROI detection (ROI-I and ROI-II), the model achieved verification levels of over 90% in both accuracy and recall. The first stage, ROI-I detection, focused on annotating maxillary tooth positions (e.g., FDI numbering) while emphasizing potential lesion areas and minimizing background noise in full-image processing. This stage laid the groundwork for more detailed subsequent analysis. The second stage, ROI-II detection, further refined the focus within the ROI-I regions, concentrating on individual tooth feature areas. This phase identified critical regions around the tooth roots and sinus cavity, achieving higher accuracy in lesion recognition. This two-stage processing workflow improved the model’s diagnostic performance and effectively reduced interference in image analysis, providing reliable technical support for clinical diagnostics.

This study utilized the YOLOv11n model and compared its performance with other traditional algorithmic models, demonstrating significant advantages in single-tooth detection tasks on DPR images. The YOLOv11n model achieved a precision and recall of 98.2% and a mAP50 of 97.8%, substantially outperforming other methods. In contrast, study [30] used Faster R-CNN and achieved a precision of only 83.8% and a recall of 79.1%, while traditional algorithmic models yielded a mAP50 of just 96.7% [31]. These results highlight YOLOv11n’s exceptional performance in detection stability and accuracy, further validating the effectiveness of the two-stage DPR detection method adopted in this study. The detection results underscore the strong potential of YOLO series models in medical imaging, particularly for small-scale, high-precision object detection tasks. Although the two-stage detection process is slightly more complex than single-stage detection, its improvements in accuracy and stability are critical. Traditional single-stage detection strategies often need help with uneven feature distribution or substantial background interference in medical images. In contrast, the two-stage detection design progressively focuses on finer feature regions, effectively filtering out unnecessary information and enabling the model to capture lesion-related features more accurately. On the other hand, the CLAHE technique used in this study proved highly effective in improving image brightness uniformity and detail visibility, significantly enhancing the model’s classification performance. In the postprocessing phase, the RGB mapping technique, which converted grayscale information into intuitive color representations, notably improved the model’s ability to identify whether tooth root apices were near the sinus floor. As shown in Table 12, the YOLOv8n-cls model achieved a classification accuracy of 96.1% after applying RGB mapping, a 4.1% improvement over DetectNet [32], and an 8% increase in recall. Furthermore, compared to traditional CNN models [33], this study achieved 20% improvement across all performance metrics, emphasizing the critical role of RGB mapping in enhancing image features. In summary, the main contributions of this study are as follows.

1. YOLO11n achieved a maximum precision of 98.2% and a recall of 98.2%. Accuracy increased by 14% compared to other models and studies, which improved by 19%.
2. The study utilized innovative odontogenic sinusitis image preprocessing and postprocessing methods to improve 16.9% accuracy in YOLOv8n-cls disease classification, which is higher than other classification models.
3. In clinical applications, YOLOv8n-cls classification accuracy for non-odontogenic sinusitis achieves 95.8%, and odontogenic sinusitis achieves 97.6%.

This study achieved several technical breakthroughs but also has certain limitations. First, the limited size of the dataset may impact the model’s generalization capability. Although data augmentation techniques partially address the issue of insufficient samples, further validation on larger-scale datasets is necessary. Additionally, during the segmentation of the ROI-II region, different models may introduce excessive irrelevant features in their processing of the tooth region, potentially affecting the stability of classification results. Future research should focus on designing more precise segmentation algorithms to minimize the introduction of noise while retaining key features relevant to the lesions. Additionally, further efforts are required to explore integrating the proposed deep learning system into existing imaging platforms used in clinical practice. This would include evaluating compatibility with current hardware and software, assessing workflow efficiency, and ensuring regulatory compliance to facilitate its adoption in real-world clinical settings.

## 5. Conclusions

This study developed an AI-assisted diagnostic system that significantly improves the accuracy of detecting and classifying odontogenic sinusitis using dental panoramic radiographs. The proposed system demonstrates strong potential for clinical applications, offering reliable support for early diagnosis and paving the way for broader intelligent dental diagnostic platforms.

## Figures and Tables

**Table 1:** YOLO11n architecture.

Block Number	Type	Kernel Size	Stride	Filters	Future Map Size
0	Conv	3	2	64	320 × 320 × 16
1	Conv	3	2	128	160 × 160 × 32
2	C3k2	-	-	256	160 × 160 × 64
3	Conv	3	2	256	80 × 80 × 64
4	C3k2	-	-	512	80 × 80 × 128
5	Conv	3	2	512	40 × 40 × 128
6	C3k2	-	-	512	40 × 40 × 128
7	Conv	3	2	1024	20 × 20 × 256
8	C3k2	-	-	1024	20 × 20 × 256
9	SPPF	5	-	1024	20 × 20 × 256
10	C2PSA	-	-	1024	20 × 20 × 256
11	Upsample	-	-	1024	40 × 40 × 256
12	Concat	-	-	-	-
13	C3k2	-	-	512	40 × 40 × 128
14	Upsample	-	-	512	80 × 80 × 128
15	Concat	-	-	-	-
16	C3k2	-	-	256	80 × 80 × 64
17	Conv	3	2	256	40 × 40 × 128
18	Concat	-	-	-	-
19	C3k2	-	-	512	40 × 40 × 128
20	Conv	3	2	512	20 × 20 × 128
21	Concat			-	
22	C3k2			20 × 20 × 256	

**Table 2:** Number of training and validation sets for object detection.

Dataset	Training Set	Validation Set	Test Set	Total
ROI-I	126	30	7	163
ROI-II	88	30	11	129

**Table 3:** ResNet18 architecture.

Block Number	Type	Kernel Size	Stride	Filters	Future Map Size
0	Conv	7 × 7	2	64	112 × 112 × 64
1	Max pool	3 × 3	2	-	56 × 56 × 64
2	Conv	3 × 3	1	64	56 × 56 × 64
3	Conv	3 × 3	1	64	56 × 56 × 64
4	Conv	3 × 3	1	64	56 × 56 × 64
5	Conv	3 × 3	1	64	56 × 56 × 64
6	Conv	3 × 3	2	128	28 × 28 × 128
7	Conv	3 × 3	1	128	28 × 28 × 128
8	Conv	3 × 3	1	128	28 × 28 × 128
9	Conv	3 × 3	1	128	28 × 28 × 128
10	Conv	3 × 3	2	256	14 × 14 × 256
11	Conv	3 × 3	1	256	14 × 14 × 256
12	Conv	3 × 3	1	256	14 × 14 × 256
13	Conv	3 × 3	1	256	14 × 14 × 256
14	Conv	3 × 3	2	512	7 × 7 × 512
15	Conv	3 × 3	1	512	7 × 7 × 512
16	Conv	3 × 3	1	512	7 × 7 × 512
17	Conv	3 × 3	1	512	7 × 7 × 512
18	Average pool	-	-	-	1 × 1 × 512
19	FC	-	-	64	1000
20	Softmax	-	-	128	1000

**Table 4:** YOLOv8n-cls architecture.

Block Number	Type	Kernel Size	Stride	Filters	Future Map Size
0	Conv	3	2	64	320 × 320 × 16
1	Conv	3	2	128	160 × 160 × 32
2	C2f	-	-	128	160 × 160 × 64
3	Conv	3	2	256	80 × 80 × 64
4	C2f	-	-	256	80 × 80 × 128
5	Conv	3	2	512	40 × 40 × 128
6	C2f	-	-	512	40 × 40 × 128
7	Conv	3	2	1024	20 × 20 × 256
8	C2f	-	-	1024	20 × 20 × 256
9	SPPF	5	-	1024	20 × 20 × 256
10	Upsample	-	-	1024	20 × 20 × 256
11	Concat	-	-	-	40 × 40 × 256
12	C2f	-	-	512	40 × 40 × 128
13	Upsample	-	-	512	80 × 80 × 128
14	Concat	-	-	-	80 × 80 × 128
15	C2f	-	-	256	80 × 80 × 64
16	Conv	3	2	256	40 × 40 × 64
17	Concat	-	-	-	40 × 40 × 64
18	C2f	-	-	512	40 × 40 × 128
19	Conv	3	2	512	20 × 20 × 128
20	Concat	-	-	-	20 × 20 × 128
21	C2f	-	-	1024	20 × 20 × 256

**Table 5:** Hardware and software platform versions.

Hardware Platform	Version
GPU	NVIDIA A100
DRAM	40 GB
**Software Platform**	**Version**
Python	3.8.13
PyTorch	1.12.1+cu116
CUDA	11.6

**Table 6:** Number of classification model training sets and validation sets.

	Training Set	Validation Set	Total
Normal	133	13	146
Odontogenic sinusitis	134	13	147
Total	267	26	293

**Table 7:** Data volume of disease symptom classification database after data augmentation.

	Original	Augmentation
Training Set	267	801
Validation Set	26	77
Total	293	878

**Table 8:** Results of two-stage single tooth detection using DPR images.

Molar Teeth Position Detection		YOLOv8n	YOLOv9n	YOLOv10n	YOLO11n
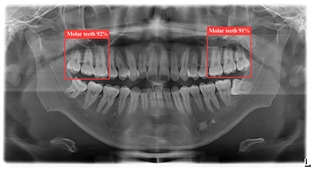	Accuracy	70.6%	89.5%	80.0%	90.0%
	Precision	92.8%	93.5%	89.9%	94.4%
	Recall	88.9%	94.4%	83.3%	94.4%
	F1 Score	90.3%	93.9%	86.4%	94.4%
	mAP50	89.7%	90.8%	83.5%	90.6%
Single Tooth Position Detection		YOLOv8n	YOLOv9n	YOLOv10n	YOLO11n
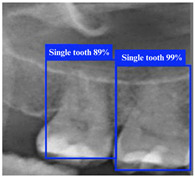	Accuracy	93.1%	88.3%	93.2%	93.2%
	Precision	96.3%	93.2%	94.8%	98.2%
	Recall	96.4%	97.6%	98.1%	98.2%
	F1 Score	96.3%	95.3%	96.3%	98.2%
	mAP50	98.5%	97.6%	98.1%	97.8%

**Table 9:** Classification model validation results with different image enhancement methods.

Method	Index	ConvNeXtv2	Swin-Transformer	ResNet18	YOLOv8n-cls	YOLO11n-cls
Original	Accuracy	54.5%	83.1%	85.7%	79.2%	90.9%
	Precision	50.0%	76.3%	81.6%	94.3%	91.4%
	Recall	74.3%	91.4%	96.5%	93.5%	88.9%
	F1 Score	59.8%	83.2%	88.4%	93.9%	90.1
Preprocessing	Accuracy	77.9%	96.1%	89.6%	91.7%	89.6%
	Precision	76.5%	93.3%	96.6%	94.3%	97.1%
	Recall	74.3%	91.4%	96.5%	93.5%	82.9%
	F1 Score	75.4%	92.3%	96.5%	93.9%	89.4%
Preprocessing + Postprocessing	Accuracy	77.9%	90.9%	94.8%	96.1%	89.6%
	Precision	69.6%	85.7%	91.4%	97.1%	97.1%
	Recall	74.3%	91.4%	96.5%	97.1%	82.9%
	F1 Score	71.9%	88.5%	93.9%	97.1%	89.4%

**Table 10:** Confusion matrix of YOLOv8n-cls validation set.

		Actual	
		No Sinus Invasion	Sinus Invasion
Predict	No sinus invasion	41	2
	Sinus invasion	1	33

**Table 11:** Clinical assessment comparison of Validation Results with Ground Truth.

Ground Truth No Sinus Invasion		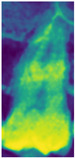	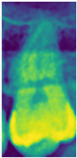	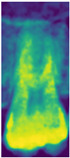	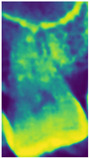	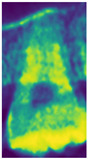	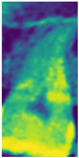	Average Precision
Precision	YOLOv8n-cls	95%	96%	95%	99%	99%	96%	97.6%
	ResNet18	89%	94%	93%	92%	94%	88%	91.7%
Ground truth Sinus invasion		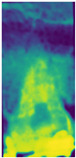	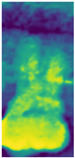	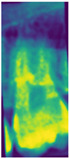	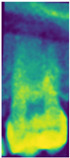	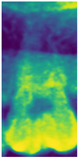	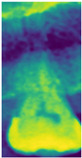	Average Precision
Precision	YOLOv8n-cls	99%	98%	95%	96%	93%	96.7%	95.8%
	ResNet18	95%	90%	87%	92%	88%	91%	9.5%

**Table 12:** Comparison with other research in sinus floor level detection.

Method	Model	Accuracy	Precision	Recall	F1-Score
This Study	YOLOv8n-cls	96.1%	97.1%	96.1%	96.6%
[32]	DetectNet	92%	96%	88%	X-
[33]	CNN Model	75.7%	75.7%	75.7%	75.7%

## Data Availability

This study will not provide any database or source code due to ethical issues.
